# TAM receptors in cardiovascular disease

**DOI:** 10.1093/cvr/cvz100

**Published:** 2019-04-13

**Authors:** Lucy McShane, Ira Tabas, Greg Lemke, Mariola Kurowska-Stolarska, Pasquale Maffia

**Affiliations:** 1 Centre for Immunobiology, Institute of Infection, Immunity and Inflammation, College of Medical, Veterinary and Life Sciences, University of Glasgow, Sir Graeme Davies Building, 120 University Place, Glasgow, UK; 2 Institute of Cardiovascular and Medical Sciences, College of Medical, Veterinary and Life Sciences, University of Glasgow, Glasgow, UK; 3 Departments of Medicine, Physiology, and Cell Biology, Columbia University Irving Medical Center, New York, NY, USA; 4 Molecular Neurobiology Laboratory, Salk Institute for Biological Studies, La Jolla, CA, USA; 5 Immunobiology and Microbial Pathogenesis Laboratory, Salk Institute for Biological Studies, La Jolla, CA, USA; 6 Rheumatoid Arthritis Pathogenesis Centre of Excellence (RACE), Institute of Infection, Immunity and Inflammation, College of Medical, Veterinary and Life Sciences, University of Glasgow, Sir Graeme Davies Building, 120 University Place, Glasgow, UK; 7 Department of Pharmacy, University of Naples Federico II, Naples, Italy

**Keywords:** TAM receptors, Tyro3, Axl, MerTK, Cardiovascular disease

## Abstract

The TAM receptors are a distinct family of three receptor tyrosine kinases, namely Tyro3, Axl, and MerTK. Since their discovery in the early 1990s, they have been studied for their ability to influence numerous diseases, including cancer, chronic inflammatory and autoimmune disorders, and cardiovascular diseases. The TAM receptors demonstrate an ability to influence multiple aspects of cardiovascular pathology via their diverse effects on cells of both the vasculature and the immune system. In this review, we will explore the various functions of the TAM receptors and how they influence cardiovascular disease through regulation of vascular remodelling, efferocytosis and inflammation. Based on this information, we will suggest areas in which further research is required and identify potential targets for therapeutic intervention.

## 1. Introduction

Insight from the results of studies using cultured cells, mouse models of cardiovascular disease subjected to genetic engineering or pharmacological intervention, and observational studies in humans have provided strong evidence that the immune responses plays an important role in cardiovascular pathologies.^1–3^ Most importantly, the CANTOS trial (Canakinumab Anti-inflammatory Thrombosis and Outcomes Study) has recently demonstrated the efficacy of targeting interleukin (IL)-1β for reducing secondary cardiovascular events,^4^ and therefore, much of current research is now focusing on how to limit inflammation to prevent cardiovascular diseases (CVD).

Among the many molecules that influence the immune response, the TAM family of tyrosine kinase receptors have demonstrated a capacity to influence the function of both the vascular and immune system in the steady state and in pathology. Accordingly, there has been much interest in their potential roles in CVD and how they may be viewed as potential therapeutic targets. This family of proteins includes Tyro3, Axl, and MerTK, with the first letters of each giving the family its name.^5^ They remained orphan receptors for the first few years following their discovery, but by the mid-1990’s their ligands were identified as growth arrest-specific 6 (Gas6) and Protein S (Pros1).^6^^,^^7^ These ligands bind to the TAM receptors with differential affinity. Gas6 can associate with all three receptors, but with strongest affinity to Axl, then Tyro3, and with lower affinity to MerTK.^6^ Pros1, however, does not bind to Axl at all, and has stronger affinity binding with Tyro3 than MerTK.^8^

## 2. TAM receptors

### 2.1 Structure

The basic structure of the TAM receptors includes an extracellular N-terminal region containing two immunoglobulin (Ig)-like domains, followed by two fibronectin type III (FNIII) domains, a hydrophobic domain which traverses the cell membrane, and finally, an intracellular tyrosine kinase C-terminal domain.^9^^,^^10^ The Gas6 and Pros1 ligands possess an approximately 50 amino acid stretch which contains gamma carboxylated glutamic acid residues, referred to as the Gla-domain. These residues have a high affinity for calcium, which facilitates binding to phosphatidylserine (PtdSer) molecules found on the surface of platelets and on the outer leaflet of cell membranes under certain conditions, notably on apoptotic cells.^11^ The C-terminus of the ligands possess laminin G (globular) domains, which facilitate their interactions with the TAM receptor Ig-like domains.^12^ As for most receptor tyrosine kinases (RTKs), activation of the TAM receptors occurs via ligand facilitated dimerization, which mediates autophosphorylation of their tyrosine kinase domain.^9^ This results in coupling of the receptor with proteins involved in signalling pathways, which will be discussed in more detail below.

### 2.2 TAM receptor functions

Remarkably, TAM receptors are not required for embryonic development, which is unusual for one RTK, let alone an entire subset.^13^ However, this property enabled the generation of viable triple TAM knock-out (KO) mice, which propelled studies exploring the functions of the TAM receptor family. When these KO mice reach adulthood, three distinct phenotypes can be observed. The first is male infertility, which occurs due to an inability to clear apoptotic gamete cells in the testes.^13^ The second phenotype is blindness due to retinal epithelial cells not being able to engulf the outer segments of the photoreceptors, which is necessary for the removal of toxic byproducts of phototransduction.^14^ The third phenotype is autoimmunity.^15–18^ This stems from the requirement to clear the apoptotic bodies generated during the immune response in order to resolve inflammation.^19^ Failure to clear dead cells can cause them to become necrotic, with their accumulation serving as a source of self-antigen.^20^

Phagocytosis of apoptotic cells (efferocytosis) is a fundamental process for the restoration of immune and tissue homeostasis,^21^ and the TAM receptors play an important role in efferocytosis in adult tissues.^22^^,^^23^ In atherosclerosis, efferocytosis is required for clearing of apoptotic cells in lesions, and in advanced atherosclerosis, this process can go awry, leading to post-apoptotic necrosis of lesional cells.^24–28^ This pathological process can lead to large areas of plaque necrosis, which are highly inflammatory and render the plaques susceptible to rupture or erosion. Ruptured or eroded plaques can then promotes occlusive vascular thrombosis, leading to acute coronary syndromes such as myocardial infarction (MI), unstable angina, sudden cardiac death, or stroke.^29^ In atherosclerosis, efferocytosis is largely orchestrated by professional phagocytes such as macrophages, which are known to express and utilize TAM receptors in this process.^30–32^ In other settings, it is likely that the non-professional phagocytes including cardiac myofibroblasts and epithelial cells also express TAM receptors,^33^^,^^34^ particularly Axl which shows high levels of expression in the heart.^35^^,^^36^

In addition to the anti-inflammatory effects of efficient efferocytosis, the TAM receptors are involved in directing the change in the immune response from attack-the-pathogen to repair-and-restore (resolution).^16^^,^^37^ This was first indicated by the phenotype of the triple TAM receptor KO mice, which develop a lymphoproliferative disorder and broad-spectrum autoimmunity driven by chronic hyper-activation of antigen presenting cells such as monocytes, dendritic cells (DCs), and macrophages.^15^

Expression of TAM receptors is up-regulated in innate immune cells upon their activation in order to prime the system for negative feedback, which is subsequently facilitated by the increased availability of their ligands upon initiation of the adaptive response.^10^ Upon ligand-mediated autophosphorylation, TAM receptors can physically associate with the type I interferon receptor (IFNAR)-STAT1 complex, which normally drives the initial amplification of inflammation. However, the association between the R1 subunit of IFNAR and the phosphorylated TAM causes a change in the function of the IFNAR-STAT1 complex to that of an anti-inflammatory signalling molecule, which in turn initiates transcription of the suppressor of cytokine signalling (SOCS)1 and SOCS3 proteins.^16^ These proteins ultimately suppress both cytokine receptor and Toll-Like receptor (TLR)3, TLR4, and TLR9 pro-inflammatory signalling pathways.^38^ This relationship between TAM receptors and ligands is key to maintaining immune homeostasis, particularly between T cells, which produce Pros1 upon activation, and DCs expressing the TAM receptors.^39^ T cell-DC interactions have been shown to occur within atherosclerotic lesions, which can enhance the pro-inflammatory nature of the plaque.^40–42^ Therefore, deregulation of TAM-mediated suppression and resolution of the inflammatory response can strongly influence cardiovascular immunity.

### 2.3 Regulation of TAM receptor expression

The expression and activity of the TAM receptors are controlled by various factors at the transcriptional, post-transcriptional, and protein levels. Induction of specific TAM receptor genes can be mediated by various cytokines. For example, transforming growth factor β (TGF-β), and granulocyte-macrophage colony-stimulating-factor, and several proinflammatory stimuli can drive Axl expression, while IL-17A, IL-10, and dexamethasone drive MerTK expression.^43–46^ As Tyro3 has not been closely studied in the context of inflammation or the immune response, it remains unclear which cytokines, if any, specifically up-regulate its expression.

Up-regulation of MerTK expression can also be induced by glucocorticoids and the liver-X-receptors (LXR) α and β.^47^^,^^48^ TAM receptor regulation by the LXRs is of interest in CVD due to their role in cholesterol biosynthesis and homeostasis, notably cholesterol efflux from macrophages.^49^

As an example of another level of regulation, microRNA-34a has been demonstrated to suppress protein expression of Axl, and this process can contribute to the autoimmune disorder rheumatoid arthritis.^50^ Similarly, microRNA-7 inhibits Tyro3 expression and is consequently being explored as an RNA-based therapeutic for treating abhorrent Tyro3 overexpression in human hepatocellular carcinoma.^51^

**Figure 1:**
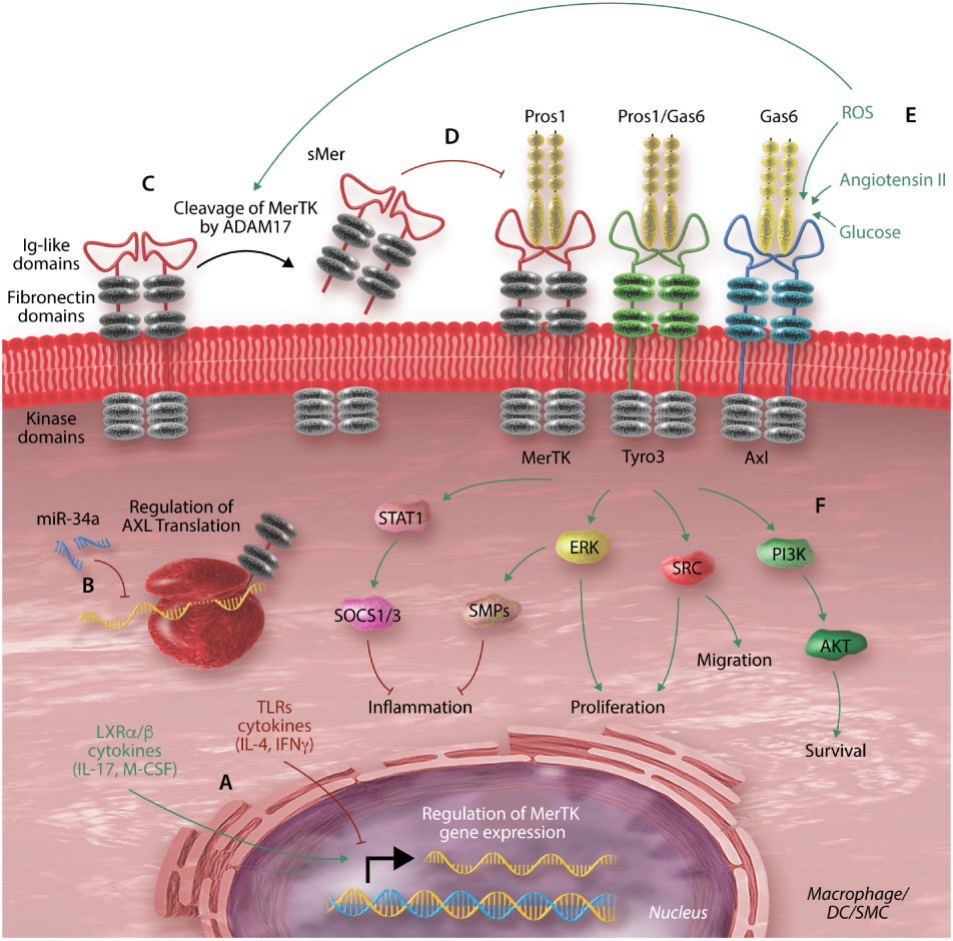
Molecular regulation of the TAM receptors. The expression and activity of the TAM receptors is controlled by various factors at the transcriptional, post-transcriptional, and protein levels. (*A*) TAM receptor gene transcription can be up- or down-regulated by various factors, including cytokines. The figure shows mediators that specifically regulate MerTK transcription; the other TAM receptors are regulated by other mediators.^43^^,^^44^^,^^46–48^ (*B*) Post-transcriptional regulation by micro-RNAs such as miR-34a inhibition of Axl expression.^50^ (*C*) At the protein level, TAM receptors are rendered dysfunctional by cleavage of their extracellular domain by metalloprotease ADAM17.^52^^,^^53^ This process which can be driven by other environmental factors such as reactive oxygen species (ROS).^53^^,^^55^^,^^56^ (*D*) The soluble byproduct released may act as a decoy for the receptor ligands, thus inhibiting TAM receptor activity.^57^ (*E*) Activation of the receptors can also be enhanced by various environmental factors.^58–60^ (*F*) Activation of the TAM receptors subsequently induces various molecular pathways affecting cell function.^16^^,^^60–62^

Once TAM receptors are fully synthesized, inserted into the plasma membrane, and activated, their extracellular domain can be cleaved by the metalloprotease A Disintegrin And Metalloproteinase (ADAM)-17.^52^^,^^53^ This cleavage liberates a soluble extracellular domain bound to ligand (sAxl or sMer) and destroys TAM receptor function. Conversely, secretory leucocyte protease inhibitor increases the expression of MerTK on the cell surface, likely by inhibiting its cleavage.^54^ The molecular regulation of TAM receptors is illustrated in *Figure 1*.

## 3. Axl in CVD

### 3.1 Gas6-Axl and regulation of cardiovascular remodelling

Vascular remodelling refers to a dynamic process that causes changes to the structure of the vascular wall. This can occur in response to certain stimuli, such as injury or local production of inflammatory mediators. In addition, chronic conditions such as *hypertension* or *atherosclerosis* can drive the process of vascular remodelling. Although vascular remodelling likely represents a response to correct environmental changes to vascular flow and maintain homeostasis, it can have pathological effects, as described below. Several vascular cell types, including endothelial cells (ECs), vascular smooth muscle cells (VSMCs), fibroblasts and myofibroblasts, contribute to vascular remodelling. Vascular remodelling occurs through four main processes: cellular migration, proliferation, survival; and extracellular matrix synthesis and degradation. For each of these processes, Axl and its ligands have been shown to play a role, while the contributions of MerTK and Tyro3 are less clear.

Both Pros1 and Gas6 were shown to be secreted by and enhance the proliferation of VSMCs prior to their identification as the ligands for TAM receptors.^58^^,^^63^ Subsequently, Axl expression was found to be increased in VSMCs following balloon-injury in rat carotid artery, and both Axl and Gas6 expression were temporally correlated with neointima formation.^64^ The key role of Gas6/Axl pathway in the regulation of vascular remodelling was confirmed in animal studies, which showed reduced intimal thickening following vascular injury in Axl^−^^/^^−^ mice compared with wild type control mice.^59^^,^^65^^,^^66^ Similarly, in the deoxycorticosterone acetate (DOCA)-salt hypertensive mouse model, Axl deficiency led to reduced systolic blood pressure^67^^,^^68^ and reduced remodelling index of the mesenteric artery.^68^ Emerging studies suggest that (i) induction of proliferation of VSMCs,^58^^,^^63^ (ii) stimulation of migration of VSMCs,^69^ and (iii) protection from apoptosis^70^^,^^71^ are underlying mechanisms linking Gas6/Axl signalling to vasculature remodelling.

Angiotensin II (Ang II) is a hormone produced as an end-product of the renin–angiotensin system and is an important mediator of numerous cardiovascular pathologies, such as hypertension, vascular remodelling, and neointima formation.^72^ Ang II has been demonstrated to activate the Gas6-Axl pathway and is necessary for facilitating the effects of Axl on VSMC proliferation.^73^^,^^74^ In a similar vein, reactive oxygen species (ROS) such as hydrogen peroxide (H_2_O_2_) elicit pathological effects on the vasculature,^75^ at least partially through Axl in VSMCs.^59^ ROS have been shown to induce interaction between Axl and glutathiolated non-muscle myosin heavy chain (MHC)-IIB, which may mediate increased migration in vascular injury,^76^ and pharmacological inhibition of Axl attenuated the pathological effects of oxidative stress and reduced VSMC migration *in vitro*.^77^ In addition, Axl appears to prolong VSMC survival, and Gas6 was shown to protect VSMCs from apoptosis.^70^^,^^71^ In this context, one study observed increased VSMC apoptosis in Axl deficient mice, leading to reduced intima-media thickening following ligation of the left carotid artery.^66^

In addition to regulating VSMC biology, activation of Axl via Gas6 exerted a mitogenic effect on serum-starved fibroblasts *in vitro* and protected these cells from cell death by apoptosis.^78^ Similarly, Gas6-Axl signalling can promote the survival and vascular endothelial growth factor A-mediated migration of ECs.^79–83^ Finally, Axl has also been shown to directly regulate cytokine/chemokine expression and extracellular matrix remodelling in the vessel wall.^84^ Axl regulates these multiple aspects of vascular function through its ability to interact with multiple downstream signalling pathways. For example, Axl-mediated effects on cell proliferation and migration are facilitated through activation of phosphatidylinositol-3-OH kinase (PI3K)/protein kinase B (Akt), sarcoma (SRC) signalling pathways, and extracellular signal-regulated kinases (ERKs), which is similar to other RTK-mediated processes.^60^^,^^61^

Remarkably, differing levels of glucose *in vitro* can affect Axl behaviour, demonstrated by the comparison of functional Axl interactions in VSMCs exposed to 5.5 mmol/L ‘low glucose’ or 27.5 mmol/L ‘high glucose’ culturing conditions.^60^ Axl was found to preferentially interact with proteins involved in the PI3K signalling pathway under ‘low glucose’ conditions, stimulating anti-apoptotic signalling and enhancing survival of the VSMCs. In contrast, Axl associated with signalling proteins of the ERK1/2 pathway in ‘high glucose’ conditions, driving VSMC migration. Ultimately, this demonstrates that the function of Axl may be altered depending on the physiological conditions.^60^ In addition to this, Axl expression is significantly lower in left internal mammary artery tissue from diabetic compared with non-diabetic patients^85^ and Axl overexpression was able to reverse the effects of high glucose-induced dysfunction in ECs *in vitro*.^85^ The precise mechanisms by which Axl signalling and function respond to varying glucose levels remains unclear. However, these findings may have important implications in terms of understanding the pathological effects of chronic high blood glucose levels in diabetic patients.

Vascular calcification, which is the process by which calcium builds up within the vasculature, is particularly prevalent in advanced, inflammatory atherosclerosis and correlates with worse clinical outcomes.^86–88^ Conversely, absence of calcification in coronary arteries predicts a low risk of CVD even in subjects with a high level of traditional risk factors.^89^ During calcification, vascular pericytes undergo a process of osteogenic differentiation, and Axl was identified as one of the genes which is down-regulated when this process was explored *in vitro*.^90^ Similarly, Axl expression was shown to be down-regulated as cultured VSMCs calcify their matrix, and Axl overexpression or activation inhibited calcification *in vitro.*^91^^,^^92^ More recently, miR-34a has been shown to promote VSMC calcification in mice and in VSMCs *in vitro*, with the *in vitro* effect of miR-34a showing a correlation with decreased Axl expression on the cultured VSMCs.^93^ Interestingly, work by another group has revealed that the ability of hydroxy-3-methylglutaryl coenzyme A (HMG CoA) reductase inhibitors (statins) to prevent phosphate-induced calcification by VSMCs *in vitro* occurs via restoration of the Gas6-Axl mediated survival pathway.^94^^,^^95^ Whether Axl affects vascular calcification *in vivo* has not yet been determined.

Axl has also been reported to be expressed by cardiomyocytes.^36^ In patients with heart failure Axl levels are amplified both in terms of the cardiac tissue and circulating soluble Axl (sAxl).^35^ Furthermore, Gas6 and sAxl levels are found to increase in patients following ST-segment elevation MI.^96^ In both studies, the levels of Axl were predictive of adverse pathology, such as the extent of left ventricular remodelling and of further cardiovascular events. One study tested the effect of both KO and cardiac-specific overexpression of Gas6 in a cardiac stress murine model.^97^ They found that Gas6-deficient mice had decreased hypertrophy, fibrosis, and contractile dysfunction in the chronic stress overload induced by aortic banding. Whereas cardiac-specific overexpression of Gas6 enhanced these pathologies. This was ultimately attributed to Gas6 activation of the ERK signalling pathway, driving cardiac hypertrophy. Interestingly, this process was reversed with the use of a pharmacological inhibitor of ERK. As discussed previously, Gas6 activation of Axl can induce the ERK signalling cascade,^98^ which combined with evidence that Axl levels are elevated in heart failure patients, points to the Gas6-Axl axis as a potential novel therapeutic target in heart failure.

In summary, multiple studies suggest that GAS6/Axl drives cardiovascular remodelling by regulating the biology of VSMCs, ECs, cardiomyocytes, and potentially fibroblasts, thereby facilitating pathological processes such as neointima formation, and remodelling in both the heart and vasculature. Most importantly, Axl suppression can dampen these adverse effects, suggesting possible therapeutic implications of these studies.

### 3.2 Gas6-Axl and inflammation in CVD

Axl performs biphasic roles in the cells of the vasculature and immune system. Axl expression has actually been suggested to drive pro-inflammatory activation of VSMCs during vein-graft remodelling.^65^ In this study, vein-graft surgeries were performed to examine the effect of Axl deficiency in both the vein graft donor and recipient mouse to compare the effect of vascular vs. systemic expression of Axl. The authors found that Axl depletion in all groups led to lower levels of MHC class II expression in the vein graft, indicating lesser immune activation. They also observed down-regulation of various pro-inflammatory cytokines and chemokines in *Axl*^−^^*/*^^−^ SMCs compared with *Axl^+/+^* with and without interferon gamma (IFN-γ) stimulation. Remarkably, they found Axl deficiency to increase the expression of SOCS1 in VSMCs, i.e., opposite to what is observed in immune cells.^10^^,^^16^^,^^50^

The transfer of Axl deficient haematopoietic myeloid cells to western diet-fed low-density lipoprotein receptor KO (*Ldlr*^−^^*/*^^−^) mice had no effect on atherosclerotic disease progression.^99^ To date no studies have examined the effect of global Axl deficiency on atherosclerosis pathology, and thus the effect of eliminating Axl expression in the vasculature has not been addressed. This remains an important point for study, as Axl has been shown to be present in human vessels with expression down-regulated in atherosclerotic plaques compared with normal carotids.^100^ Furthermore, Axl expression is higher in vessels that are less prone to develop atherosclerosis such as the left internal mammary artery compared with the aorta.^101^ Concomitantly, elevated serum levels of sAxl have been detected in the setting of acute coronary syndromes.^102^ The Axl ligand Gas6 is expressed by ECs, VSMCs, and macrophages and its expression increases with atherosclerosis development.^103^^,^^104^ Genetic KO of Gas6 increases plaque stability in *ApoE*^−^^*/*^^−^ mice, leading to increased plaque content of SMCs and collagen and to reduced numbers of macrophages.^105^ However, data from an epidemiological studies showed that low levels of Gas6 correlate with increased risk of coronary artery disease (CAD) in patients with psoriasis.^106^

An interesting study used chimeric mice with haematopoietic or non-haematopoietic Axl deficiency to dissect the role of Axl expression in immune vs. vasculature cells in hypertension.^67^ The data indicated that Axl-expressing immune cells drove pro-inflammatory gene expression and increased immune cell infiltration in the kidney at early stages of hypertension and that Axl expression in both immune and vascular cells was detrimental in the later phases of hypertensive disease. Continuing on from this, the same group demonstrated that Axl is critical for survival of T lymphocytes, affecting vascular remodelling and inflammation in DOCA-salt induced hypertension.^107^

Axl has been suggested to influence natural killer (NK) cell development. However, this conclusion is controversial, with studies supporting a role for Axl in both the suppression and promotion of NK cells.^108–110^ Although none of these studies are focused on cardiovascular disease, this could be an interesting area of future study. On the one hand, NK cells may have a potentially protective role in CAD based on strong correlations between coronary heart disease and low NK levels in humans.^111^^,^^112^ However, animal studies suggest that NK cells are atherogenic.^113^ The role of Axl in NK cells and the role of NK cells themselves in cardiovascular disease represent areas that will benefit from further studies, which is necessary for a better understanding of Axl’s role in vascular pathology and its potential as a therapeutic target.

## 4. MerTK in CVD

### 4.1 MerTK-mediated efferocytosis in atherosclerosis

MerTK deficiency leads to enhanced pathology in mouse models of atherosclerosis.^30^^,^^32^ One study backcrossed MerTK KOs into *ApoE*^−^^*/*^^−^ mice, which were maintained on western diet for either 10 or 16 weeks to examine the differential effect of MerTK deficiency on early or advanced lesions.^32^ The pathological effects of MerTK-deficiency were apparent in advanced, as opposed to early, lesions owing to a marked decrease in the clearance of apoptotic bodies and the subsequent accelerated plaque necrosis. Interestingly, the study found no apparent effect on overall lesion size or plasma cholesterol profile, despite other studies showing that effective efferocytosis is beneficial early on in lesion development.^25–27^ Thus, other phagocytic receptors may play a role in efferocytosis in early atherosclerosis, although Axl is not likely among these receptors, as mentioned above, the transfer of Axl deficient bone marrow cells to *Ldlr*^−^^*/*^^−^ mice had no effect on atherosclerotic disease progression.^99^

The effects of MerTK in atherosclerosis progression to date has been investigated in phagocytic immune cells—primarily macrophages. This can be inferred from a study which utilized a chimeric mouse model, in which bone marrow cells deficient in MerTK were transferred into *Ldlr*^−^^*/*^^−^ mice.^30^ Similar to the previous study, MerTK deficiency lead to significantly higher levels of apoptotic debris accumulation within the plaque after 15 weeks of western diet. The authors were also able to show increased inflammation in the lesions of these mice. In this study, there was a 60% increased lesion size in the *Mertk*^−^^*/*^^−^*Ldlr*^−^^*/*^^−^ mice compared with *Mertk^+/+^ Ldlr*^−^^*/*^^−^. It is possible the differences in effect on lesion size may be due to differences between the mouse models and/or composition of the diets used in the two studies. More recently, a macrophage Ca^2+^/calmodulin-dependent protein kinase IIγ (CaMKIIγ) pathway was shown to play a key role in the development of necrotic atherosclerotic plaques by preventing MerTK expression through the inhibition of the transcription factors ATF6 and LXRα.^114^

MerTK is expressed in macrophages in human atherosclerotic arteries,^100^ and MerTK induction is required for clearance of apoptotic cells by human macrophages.^115^ Interestingly, it has been suggested that macrophage MerTK deficiency can occur near the necrotic cores of human plaques via the action of ADAM17-mediated MerTK cleavage.^55^ ADAM17 can be activated by the byproducts of polyunsaturated fatty acid oxidation and by inflammatory mediators, which are known to be present within the necrotic core of atherosclerotic plaques.^53^^,^^55^ Levels of sMer within individual plaques were shown to correlate with the extent of necrosis, and mice expressing genetically modified MerTK resistant to cleavage (MerTK^Δ483-488^) showed improved lesional efferocytosis, more stable plaques, and, interestingly, improved resolution of inflammation^31^ (see below).

Although research in this area has mainly focused on the role of MerTK in macrophages, it should also be noted that brain microvascular ECs have been shown to express MerTK,^116^ and that it is required for tightening the blood–brain barrier during viral infection.^117^ In atherosclerosis, inflammation and oxidative stress can destabilize the endothelial barrier, contributing to pathology.^118^ Therefore, MerTK, by maintaining the integrity of the endothelial barrier, may in principle also contribute to impeding the development of atherosclerosis; however, further work needs to be done to explore this aspect.

### 4.2 MerTK-mediated efferocytosis in myocardial infarction

Another important facet of cardiovascular pathology that relies on efficient efferocytosis, includes the clearing of dead cardiomyocytes following MI.^119–121^ This process is orchestrated by various immune cells, and the inflammatory profile of these cells can have a major influence on the functional outcome and subsequent progression of heart failure.^122^ MerTK-expressing monocyte/macrophages are key for the clearance of injured cardiomyocytes and improve remodelling following MI in mice.^123^ Conversely, genetic deficiency of MerTK led to an increase in the accumulation of apoptotic cardiac cells following experimental MI, resulting in larger infarct sizes and worse cardiac functional outcomes.^124^ Cardiac extracts from the *Mertk^+/+^* control mice showed the presence of sMer following MI, which is likely due to the presence of post-MI ADAM17-activating factors that promote MerTK cleavage.^125^

In a study investigating adverse effects of post-MI ischaemia–reperfusion (IR) in mice and humans using therapeutic interventions to restore blood-flow, such as coronary stents and thrombolytic agents,^126^^,^^127^ serum levels of sMer were found to be elevated.^56^ In the mouse models, this finding correlated with lower expression of intact MerTK on the surface of cardiac macrophages. The study went on to show that mice possessing genetically modified cleavage-resistant MerTK (MerTK^Δ483-488^) displayed improved levels of efferocytosis, reduced infarct size, and improved cardiac function following IR. It was concluded that the crucial role of MerTK in facilitating phagocytic clearance of cardiac debris following MI was hindered by IR-induced MerTK cleavage. They hypothesized that the trigger for this was recruitment of monocytes from the circulation, which they tested by treating mice with an antagonist to block C-C chemokine receptor type 2 (CCR2), a chemokine receptor expressed by infiltrating circulatory monocytes, but not in cardiac-resident macrophages. This treatment improved post-IR infarct size in *Mertk^+/+^* but not *Mertk*^−^^*/*^^−^ mice, suggesting that CCR2-mediated infiltration negatively affects ability of MerTK to effectively drive phagocytic repair following IR. The exact mechanism for CCR2-dependent MerTK cleavage is still ambiguous. One suggestion is activation of ADAM17-mediated cleavage of MerTK by CCR2^+^ monocyte production of ROS.^53^^,^^55^^,^^56^

Interestingly, dying cardiomyocytes in the setting of MI promote the release of MerTK from the surface of macrophages, which prevents engulfment of dying cardiomyocytes.^125^ The mechanism involves up-regulation on cardiomyocytes of the ‘don’t-eat-me’ marker CD47 following MI.^128^ Although this process during MI is pathologic, as uncleared apoptotic cardiomyocytes drive cardiac inflammation and further cardiomyocyte death, it should be noted that in non-pathological conditions, evading efferocytosis may preserve cardiomyocyte numbers in view of their normally low regenerative capacity. In the pathologic setting of MI, treatment with anti-CD47 subsequently enhanced cardiomyocyte phagocytosis and reduced infarct size.^128^ Accordingly, further investigation into the mechanism by which cardiomyocytes facilitate MerTK shedding and how this can be prevented may present a novel target for therapeutic intervention in improving post-MI recovery.

### 4.3 MerTK mediated resolution of inflammation in atherosclerosis

The MerTK studies in the *Apoe*^−^^*/*^^−^ and chimeric *Ldlr*^−^^*/*^^−^ atherosclerosis models found a heightened inflammatory pathology associated with loss of MerTK.^30^^,^^32^ Ait-Oufella *et al.* showed that *ex vivo* cultured splenocytes from *Mertk*^−^^*/*^^−^ mice had increased production of pro-inflammatory cytokines IFN-γ, IL-12, and tumour necrosis factor (TNF)-α and decreased production of anti-atherogenic IL-10, showing this phenotype to be inherent in these cells and not just a consequence of the defective apoptotic debris clearance. Mice expressing cleavage-resistant MerTK generate increased levels of specialized pro-resolving mediators such as TGF-β and IL-10 and lipid mediators such as resolvins in atherosclerotic lesions,^31^^,^^37^ which, together with enhanced efferocytosis, contribute to attenuation of cardiovascular pathology in atherosclerosis. In terms of lipid mediators, when MerTK engages a ligand, a particular tyrosine residue in the cytoplasmic tail of MerTK signals inhibition of a calcium/CaMKII pathway that normally favours the biosynthesis of long-chain unsaturated fatty acid-derived inflammatory mediators, notably leukotrienes, over resolution mediators such as lipoxins and resolvins.^37^^,^^62^ Thus, MerTK signalling increases the ratio of pro-resolving-to-pro-inflammatory lipid mediators.

Activation of LXR expression occurs as a result of apoptotic cell clearance, particularly in the presence of excess-lipoprotein derived cholesterol, as would be expected within advanced atherosclerotic lesions.^129^ This process has been shown to promote expression of MerTK, which mitigates pro-inflammatory cytokine release upon subsequent exposure to cholesterol-loaded apoptotic macrophages.^24^^,^^130^ In addition, human protein S inhibits the expression of macrophage scavenger receptor A through MerTK, leading to reduced uptake of modified lipoproteins.^131^ Therefore, MerTK appears to sit at the interface between lipid metabolism and inflammation within the plaque and functions to attenuate inflammation in this environment.

## 5. Tyro3 in CVD

Apart from a study showing an association between Tyro3 and MerTK variants and carotid atherosclerosis,^132^ the net contribution of Tyro3 to CVD has not been addressed to date. This may be because Tyro3 is mainly localized to the central nervous system and reproductive organs, and doesn’t show high levels of expression in the vasculature.^36^^,^^133^^,^^134^ It’s ligand Pros1 has been found to correlate with coronary heart disease risk and is expressed in atherosclerotic lesions.^100^^,^^135^

Interestingly, Tyro3 is believed to negatively regulate T helper type 2 (Th2) cells via the suppression of a specific subset of CD11c^+^ DCs expressing programmed cell death protein 2 (PDL2).^136^ PDL2^+^ DCs are associated with driving Type 2 (Th2-driven) immune responses, and expression of Tyro3 in these cells was shown to decrease their Th2-associated molecule production. This pathway functions as part of a negative feedback loop, whereby Th2-associated cytokine IL-4 induces sustained expression of Pros1, which then activated Tyro3-mediated suppression of Th2 activation. In terms of relevance to CVD, while IL-4 and IL-5 have been shown to be atheroprotective,^137^^,^^138^ IL-9 may be pro-atherogenic.^139^ IL-13 production exerts adverse effects on cardiovascular pathology by driving fibrosis of heart tissue in the context of both ageing and IR injury,^140–142^ but IL-13 secreted by regulatory T cells may actually promote efferocytosis in atherosclerotic lesions.^143^ These combined observations provide a strong rationale for future studies on the role of Tyro3 in CVD.

## 6. Conclusions and future questions

**Figure 2:**
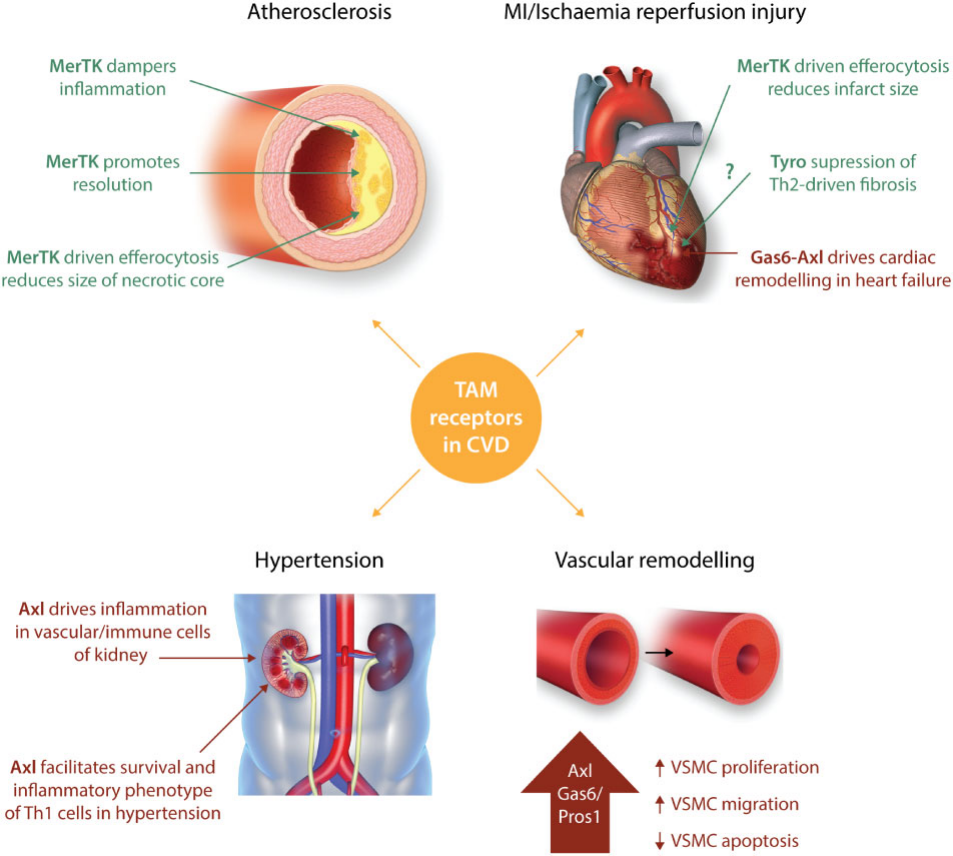
Roles of TAM receptors in various cardiovascular diseases. Pathological roles for TAM receptor family members in cardiovascular disease are shown in red, with protective roles depicted in green. MerTK-deficiency has been shown to be detrimental in atherosclerosis models owing to its ability to dampen inflammation, promote resolution, and drive clearance of apoptotic bodies in the plaque necrotic core.^30^^,^^32^^,^^56^^,^^130^ These processes can be inhibited by MerTK cleavage, which occurs in necrotic, inflammatory plaques.^31^^,^^55^^,^^56^ In hypertension models, Axl expression in both vascular and immune cells has been implicated to drive pro-inflammatory responses in the kidney,^67^ and to affect T cell survival, vascular inflammation and remodelling.^107^ A major contribution to heart failure in coronary heart disease is due to tissue damage and fibrosis following myocardial infarction. Efficient clearing of dead cardiomyocytes is crucial for restoration of cardiac function, and MerTK has been shown to play a protective role in this setting.^124^ This process can be hindered by cleavage of MerTK, which is increased following ischaemia–reperfusion.^56^ Although Tyro3 could potentially have a protective effect on the myocardium, as it suppresses Th2 responses which drive cardiac fibrosis,^136^^,^^140–142^ a direct causal link has not been shown to date. Gas6-Axl driven activation of the ERK signalling cascade in cardiomyocytes is implemented in the pathological remodelling which occurs in heart failure patients.^35^^,^^96^^,^^97^ Numerous studies have highlighted Axl to also have a pathological role in vascular remodelling through increasing VSMC proliferation, migration, and immune activation, while also inhibiting VSMC apoptosis.^64^^,^^65^^,^^69^^,^^79^

The TAM receptors—particularly MerTK and Axl—appear to have very different roles in suppressing or driving elements of cardiovascular pathology (*Figure 2*). In terms of what is currently known, MerTK is fundamentally protective in its role. This is mediated by distinct but complementary functions: suppression of the inflammatory response, enhancing the inflammation resolution response, and facilitating the clearance of apoptotic cell debris. By these criteria, as well as the protective role of MerTK in the heart itself, enhancing MerTK synthesis or functioning, and/or blocking its cleavage, may represent novel therapeutic approaches to CVD. However, this approach must consider the possible adverse effects of heightened MerTK function on cancer^144^^,^^145^ and possibly pathologic liver fibrosis.^146^

There is controversy within the literature in terms of a protective vs. a detrimental role of Axl in CVD. This largely stems from apparent differences in Axl function, particularly in terms of activating inflammation and the cell type in which it is expressed. While there is a molecular signalling pathway by which Axl has been shown to suppress the immune response, the role of this anti-inflammatory pathway in vascular cells is controversial.^16^^,^^65^ On the other hand, Axl signalling may be protective against vascular calcification.^90^^,^^91^^,^^95^ Therefore, future work will be required to sort out the mechanisms that facilitate Axl’s opposing roles in vascular and immune cell types and how these roles affect overall cardiovascular pathology.

Tyro3 remains to be the least studied of the TAM receptors, particularly in the area of cardiovascular disease. Other than one study which found an association of a Tyro3 SNP in carotid atherosclerosis^132^ we could find no published work to date investigating the role of Tyro3 in the context of cardiovascular pathology. However, Tyro3 may possess a protective function in its ability to suppress of type 2 immune responses, which promotes cardiac fibrosis.^140–142^ Thus, with further work in this area, it is possible that targeting Tyro could be considered as a fibrosis-preventing therapy post-cardiac injury.

Finally, there are studies suggesting the Gas6/TAM signalling pathway is essential for platelet activation and thrombus stabilization. For example, mice deficient in Gas6 or TAM receptors or treated with inhibitors of Gas6/TAM receptor pathways develop less arterial and venous thrombosis. The GAS6/TAM receptor role in haemostasis and thrombosis has been recently fully reviewed elsewhere^147^ and is therefore not the focus of the current review. Nonetheless, this role of TAM receptors needs to be considered in view of the key contribution of thrombosis to atherosclerosis and its clinical complications.

**Conflict of interest:** none declared.

## Funding

This work was supported by the British Heart Foundation [PG/12/81/29897 to P.M., RE/13/5/30177, and FS/16/55/32731]; the Engineering and Physical Sciences Research Council (EPSRC) [EP/L014165/1 to P.M.]; the European Commission Marie Skłodowska-Curie Individual Fellowships [661369 to P.M.]; the Arthritis Research UK [RACE20298 to M.K.S.]; and National Institutes of Health [HL075662, HL127464, and HL132412 to I.T.].

## Notes

This article was handled by a Consulting Editor, Ziad Mallat.
